# Sustaining the future of HIV counselling to reach 90-90-90: a regional country analysis

**DOI:** 10.7448/IAS.19.1.20751

**Published:** 2016-05-13

**Authors:** Marielle Bemelmans, Saar Baert, Eyerusalem Negussie, Helen Bygrave, Marc Biot, Christine Jamet, Tom Ellman, Amanda Banda, Thomas van den Akker, Nathan Ford

**Affiliations:** 1 Médecins Sans Frontières, Analysis and Advocacy Unit, Operational Centre Brussels, Brussels, Belgium; 2 Médecins Sans Frontières, Southern Africa Medical Unit, Cape Town, South Africa; 3 World Health Organization, Department of HIV/AIDS, Geneva, Switzerland; 4 Médecins Sans Frontières, Operations Department, Operational Centre Geneva, Geneva, Switzerland; 5 Department of Obstetrics, Leiden University Medical Center, Leiden, Netherlands

**Keywords:** adherence support, antiretroviral therapy, community health workers, HIV/AIDS, HIV testing and counselling, human resources for health, lay counsellors, lay workers, patient education and counselling, retention in care, sub-Saharan Africa, task shifting

## Abstract

**Introduction:**

Counselling services are recommended by the World Health Organization and have been partially adopted by national HIV guidelines. In settings with a high HIV burden, patient education and counselling is often performed by lay workers, mainly supported with international funding. There are few examples where ministries of health have been able to absorb lay counsellors into their health systems or otherwise sustain their work. We document the role of lay cadres involved in HIV testing and counselling and adherence support and discuss approaches to sustainability.

**Methods:**

We focused on a purposive sample of eight sub-Saharan African countries where Médecins Sans Frontières supports HIV programmes: Guinea, Lesotho, Malawi, Mozambique, South Africa, Swaziland, Zambia and Zimbabwe. We reviewed both published and grey literature, including national policies and donor proposals, and interviewed key informants, including relevant government staff, donors and non-governmental organizations.

**Results and discussion:**

Lay counsellors play a critical role in scaling up HIV services and addressing gaps in the HIV testing and treatment cascade by providing HIV testing and counselling and adherence support at both the facility and community levels. Countries have taken various steps in recognizing lay counsellors, including harmonizing training, job descriptions and support structures. However, formal integration of this cadre into national health systems is limited, as lay counsellors are usually not included in national strategies or budgeting.

**Conclusions:**

The current trend of reduced donor support for lay counsellors, combined with lack of national prioritization, threatens the sustainability of this cadre and thereby quality HIV service delivery.

## Introduction

By mid-2015, antiretroviral therapy (ART) was scaled up to reach 15 million people [1]. The number of people on ART is set to increase even further as new evidence around the benefits of early treatment [2,3] has led to changes in World Health Organization (WHO) guidelines, which now recommend treatment for every person with a confirmed HIV diagnosis irrespective of disease status [4]. The latest 90-90-90 global HIV targets aim to maximize the individual and population benefits of HIV treatment, by ensuring that, by 2020, 90% of people living with HIV (PLHIV) know their HIV status, 90% of those diagnosed with HIV are on ART and 90% of those on ART achieve viral suppression [5]. However, despite increases in HIV testing rates, more than half of the estimated 35 million PLHIV do not know their HIV status and less than half of those diagnosed with HIV are linked to any sort of HIV care [6]. In low- and middle-income countries, one-third of patients are lost to care within three years and of these almost 40% will have died [7,8].

These losses in the HIV care and treatment cascade have prompted programmes and health systems to implement strategies to identify HIV-positive individuals and retain them in care. Expanding entry points for HIV testing by scaling up both provider-initiated HIV testing and community-based testing strategies may augment the number of people who are aware of their HIV status, including those with higher CD4 counts, and improve linkage to care [9]. Patient education, particularly counselling and peer support, have shown positive results with regard to access to and retention in HIV care, as well as adherence to treatment [10–16].

In many countries, these patient education and counselling tasks are primarily performed by lay counsellors, who are trained for these tasks without acquiring a formal professional or paraprofessional certificate or degree [17] and who may or may not be HIV positive themselves. In some settings, such lay counsellors perform a wider range of tasks such as home-based care and additional support to the wider health system, in the form of screening, patient referral, drug refills and administrative tasks [18]. The WHO and others have recognized the potential for lay counsellors to improve clinical and public health outcomes in the HIV response, in particular by supporting HIV testing services (HTS), improving adherence and enhancing community-based ART delivery [16,19–22]. Documented benefits of these cadres include shorter waiting times for patients, improved adherence and retention in care and a reduction of the burden on the health system by decreasing the workload of professional staff [18,23–31]. Counselling is essential in order to secure minimal losses along the cascade.

Because there is a critical shortage of professional health workers in sub-Saharan Africa as well as an unequal distribution between urban and rural areas, in many places certain activities have been task-shifted to cadres with less professional training, in this case lay counsellors [32,33]. Lay counsellors can be scaled up relatively quickly due to shorter training time and a lower salary package compared to professional health staff.

This review focuses on the role of health facility-based lay counsellors involved in patient education and counselling for HIV. Although lay counsellors are widely mobilized in HIV programmes, job profiles, formal recognition, supervision and training support are often non-existent, and salary scales vary widely [16,18,34,35], despite regular calls for streamlining such support by the WHO [19,33,36]. Lay counsellors are usually employed by different implementing organizations, which results in a lack of cohesion and sustainability. For example, in Lesotho there was a reduction of lay counsellors from 487 in 2011 to 165 in 2013 following a decrease in funding. Over the same period, facility-based HTS decreased by 15%, from 253,994 tests in 2011 to 215,042 in 2012 [37].

We reviewed the provision of HTS and adherence support by lay counsellors across eight countries in sub-Saharan Africa where Médecins Sans Frontières (MSF) supports programmes, in order to map out the contribution of lay counsellors to the HIV response across a range of countries.

## Methods

Country selection was based on a purposive sample of countries with a high HIV burden where MSF has been supporting the Ministry of Health (MOH) to deliver HIV programmes at the district or national level: Guinea, Lesotho, Malawi, Mozambique, South Africa, Swaziland, Zambia and Zimbabwe. MSF's assistance includes technical support, innovation in service delivery and capacity building around HIV services scale-up, in particular ART, and addressing gaps in the HIV treatment cascade.

Information was collected between November 2014 and August 2015. A rapid literature review in MEDLINE and Google Scholar was carried out up to June 2015 to identify studies reporting on the role and impact of lay counsellors in sub-Saharan Africa using the following search terms: *lay counsellors*, *lay workers*, *ART*, *HIV testing and counselling*, *community health worker (CHW)*, *patient education and counselling*, *retention in care and task shifting*. In addition, international reports from UNAIDS, the WHO and the World Bank, as well as relevant national guidelines, policy documents and strategic plans from the MOH and other ministries, donors and non-governmental organizations (NGOs) at the country level were reviewed.

Between eight and ten interviews were conducted in each study country by in-country-based MSF staff that had been engaged in that specific country for at least two years and were familiar with the context. A purposive sample was used for the interviews. Key informants were selected among the MOH and other ministries (finance and public service), in-country donor representatives including those of the President's Emergency Plan for AIDS Relief (PEPFAR), the Centers for Disease Control and Prevention (CDC) and staff from UNAIDS, the WHO and several NGOs. In addition, 10 interviews took place with representatives from seven international donors and organizations’ head offices. Of those organizations, key informants were selected based on their work experience in Human Resources for Health (HRH) and/or HIV/AIDS services and more particularly their involvement in management or implementation of counselling services and lay counsellor programmes. A standardized semi-structured interview guide was developed. Findings of the interviews were analyzed by the study team using a thematic analysis. Following the key questions and themes that arose during the interviews and literature review, the main topics and trends were drawn out. Where possible, primary data obtained through the interviews and secondary data obtained through the document review were triangulated to validate the findings.

Results were validated by several feedback mechanisms, which also served to address potential bias due to the study team nearly solely consisting of MSF staff. Firstly, the findings were shared with the respective MOH and funding and implementing partners to seek further feedback and clarification during in-country face-to-face interviews. Secondly, a consultation on the role of lay counsellors in HIV testing and adherence support was organized jointly by the WHO and MSF in Johannesburg in April 2015. The meeting was attended by 40 senior representatives from ministries of health, finance and public service, as well as participants from civil society organisations and donors from the Southern African region. The study findings were shared and corrections or clarifications were made by participants. Experiences and approaches for improving recognition and sustained support for counsellors were summarized in an MSF institutional report published in 2015 [38]. Lastly, a team of government, donor and NGO partners provided further comments on early drafts of the findings and conclusions. Selection of these was based on a purposive sample of representatives working both at the country and head office levels in the fields of HIV, HRH and where possible with a specific focus on HTS and adherence counselling. Names are provided in the acknowledgement section.

## Results

**Figure 1:**
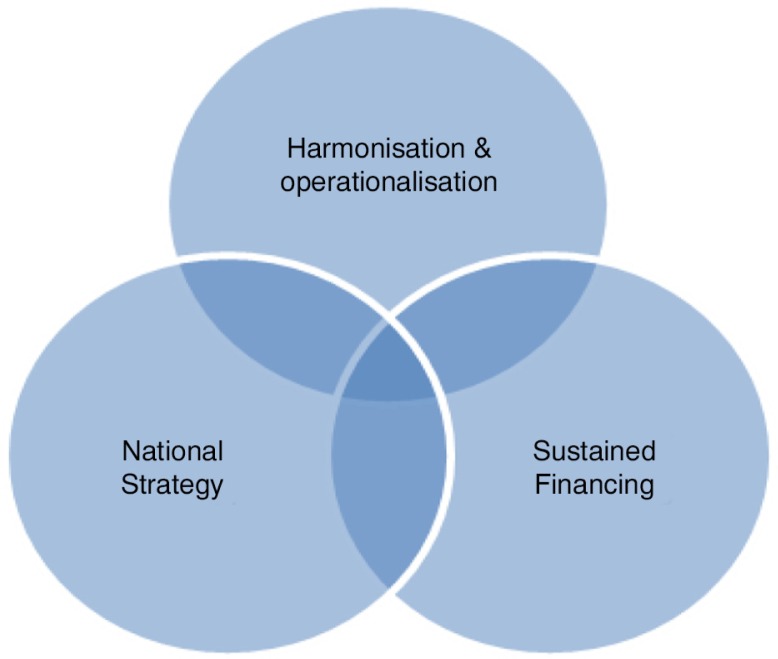
Recognition framework for lay counsellors [38].

Informal shifting of counselling-related tasks has happened in a variety of settings in sub-Saharan Africa without formal support from the MOH or NGOs [39]. A higher level of acceptance of task shifting HTS and adherence counselling interventions has been reported in other settings, such as Lesotho, Mozambique and South Africa, mainly through NGO-supported programmes, but also lacking formal recognition or coordination by the MOH. Based on country experiences [38] supplemented by a review of the literature [40,41], three components were identified by the study team as necessary to the process of realizing formal recognition of lay counsellors: (i) progress in harmonization of approaches; (ii) clear national strategies supporting lay counsellors and (iii) sustained financing (Figure 1 and Table 1).

**Table 1:** Strategies and financing for HIV counselling [38]

	Guinea	Malawi	Mozambique	Lesotho	South Africa	Swaziland	Zambia	Zimbabwe
Job title	Conseiller/médiateur (“counsellor” or “mediator”)	HSA	Lay counsellor	Lay counsellor	Lay counsellor	(Lay) HTS counsellor (two levels)	Psychosocial counsellor	Primary counsellor
Job profile and tasks	Not harmonized	Set job profile with generalized tasks: variety in primary healthcare, e.g. health promotion, immunization and other preventive activities	Set job profile but not implemented and highly variable, depending on supporting partner	Set job profile	Job profile varies by province, HIV/TB specialized	Job profile agreed, HIV/TB specialized	Set job profile, HIV/TB specialized	Set job profile, HIV/TB specialized
HTS		Yes	Yes	Yes	Yes	Yes	Yes	Yes
Adherence counselling		No	Yes	Yes	Yes	Yes	Yes	Yes
Health education		Yes	Yes	Yes	Yes	Yes	Yes	Yes
Paediatric disclosure counselling		No	Yes	Yes	Yes	Yes	No	Yes
Facilitation of community-supported models of ART delivery		No	Yes	No	No	No	No	No/in progress
Other primary health care tasks		Yes	No	Yes	Yes (administrative, data filing)	Yes (filing, pre-packing drugs)	No	No
Numbers	Unknown	Target 1/1000 population, but 10,073 available=0.63/1000	Target is 1893 countrywide but only approximately 500 currently present=0.02/1000	Target is two to three per health centre but number was reduced from 487 to 165 due to lack of funding. Now 540 proposed by GFATM=0.25/1000	72,000 CHWs, including lay counsellors countrywide=1.4/1000	Approximately 455 available	Two to three per health centre	One per health centre
Training	No standardized training	Twelve weeks basic+three weeks HTS Approximately 96% trained	HTS: two weeks two months agreed modules for HTS and adherence counselling but in reality great variety depending on supporting partner	Two weeks	Ten days HTS+additional modules	Two weeks classroom, six weeks practical	Eight weeks	Six months (including three weeks classroom) – recently increased to nine months
Supervision	Lack of regular supportive supervision	Senior HSA Nurse/clinician in charge	Psychologist or professional counsellor	Nurse/clinician in charge or senior counsellor	Counsellor supervisor and/or nurse/clinician in charge	Clinician/nurse in charge and national expert client coordinator	Nurse/clinician in charge, professional counsellors	Nurse in charge
Staff establishment	Not absorbed	4000 not yet absorbed, but all (apart from 500) government-funded	Not absorbed – wage bill and administrative issues	Not absorbed – wage bill and administrative issues	Apart from some provinces (e.g. Eastern Cape), not absorbed – to avoid paying minimal civil servant package	Not absorbed	Absorbed, but limited in number (800 to 1000 out of 40,000 trained)	Not absorbed
Remuneration (USD per month)	340 to 375	115	117 proposed 40 to 250, depending on supporting partner	70 MOH defined	90 to 335; varies by province	360 to 550; varies by partner	500 MOH defined 100 to 300, varies per partner	220
Financing	100% partner	95% government	Nearly 100% donor/partner	100% donor/partner	Most provincial budgets with some donor funds	100% donor/partner funded	800 to 1000 paid by the MOH, remaining by partner/donor	100% donor (mainly GFATM)

ART, antiretroviral therapy; CHW, community health worker; HSA, health surveillance assistant; HTS, HIV testing services; GFATM, Global Fund to fight AIDS, Tuberculosis and Malaria.

### Progress in harmonization of approaches

Harmonized training curricula, job profiles, structures for supervision and remuneration packages have been defined by the MOH and NGOs in Lesotho, Mozambique and Zimbabwe. The MOH provides certification for HTS training in Lesotho, Mozambique, Zambia and Zimbabwe. In all assessed countries, lay counsellors have been mainly deployed to enable HTS scale-up. The in-country interviews indicated that their role had expanded to include HIV and TB adherence counselling, although this is not yet reflected in training curricula, which remain mostly focused on HTS with limited guidance on adherence counselling.

Apart from Malawi and Guinea, all countries had a specific facility-based cadre dedicated to HIV-related counselling and patient education. However, in reality these cadres often combined core activities with other support services, such as administrative tasks and medication refills.

In Malawi, HTS tasks were integrated into the job profile of health surveillance assistants (HSAs), a recognized CHW cadre. Despite being a community cadre, the scope of practice for HSAs includes additional basic health checks and health promotion at the health facility level. They spend approximately half of their time in the community and the other half at the facility.

Regarding community counselling needs, Zambia trained and deployed specific community-based counsellors (community health assistants) for these tasks, whereas other countries integrated HTS tasks within the job profile of existing community cadres, such as the village health workers in Lesotho and CHWs in South Africa.

In Malawi, Lesotho, South Africa, Swaziland, Zambia and Zimbabwe, lay counsellors must complete secondary school, though most lay counsellors in place have not been educated up to this level. They are mostly members of the local communities, facilitating links between health facility and community, such as performing defaulter tracing or home-based care support. Recruitment of lay counsellors among PLHIV takes place in Swaziland and some places in Guinea.

There are different levels and structures for supervision of lay counsellors observed among countries, and in all settings it is reported to be limited and irregular due to competing priorities. In Malawi, senior HSAs are in charge of supervision, whereas in Zimbabwe and South Africa, the nurse in charge of the health facility supervises the lay counsellors. In Zambia, supervision and training of lay counsellors is accomplished through a cadre of professional counsellors. Lay counsellors can also request professional counsellors for assistance with patients requiring more complex counselling needs.

In the majority of assessed countries, international donors such as PEPFAR, the Global Fund to fight AIDS, Tuberculosis and Malaria (GFATM) and NGOs have financed lay counsellors thus far. Despite the MOH fixing the amount for monthly salaries in some countries, for example Lesotho at 70 USD and Zambia at 500 USD, it cannot enforce such measures because lay counsellors are not part of their funded staff. In reality salaries are strongly dependent on the supporting NGO and may vary by three or four times within a country (Table 1).

#### National strategies supporting lay counsellors

National guidelines defining quality criteria for counselling and patient education are generally available in the assessed countries for HTS. However, countries generally do not have national guidelines outlining adherence support standards, apart from South Africa, Zimbabwe and Mozambique. South Africa has recently developed an integrated adherence guideline for HIV/TB and non-communicable diseases, which is about to be released. Guidance on the role of Zimbabwe's lay counsellors, named “primary counsellors,” and standards of patient counselling and education are presented in their operational and service delivery manual for HIV care [42]. Mozambique's national ART scale-up plan has included a target number of lay counsellors of 1893 in 1414 health facilities, required to achieve national targets [43]. The MOH and NGOs in Lesotho require two or three lay counsellors per health facility [44]. Swaziland is the only country that has developed a specific task shifting framework that includes the scope of practice for certain cadres, their supervision and remuneration. Implementation of this framework is limited due to lack of resources. In Guinea, NGOs have task shifted patient education and adherence counselling to trained volunteers, but this is not integrated in national policy. All countries lack a professional body overseeing and protecting the work of counsellors. This contrasts with other professional health councils such as nursing associations. Only Zambia has a specific counselling council, which assisted in the creation of the psychosocial counsellors’ cadre, previously lay counsellors. Through the council's support, psychosocial counsellors became recognized and part of the country's formal workforce.

### Options for sustained financing

Four examples of contracting and financing interventions were identified. Table 2 summarizes the advantages and disadvantages of each option.

**Table 2:** Contracting and financing options [38]

Contract types	Advantages	Disadvantages	Countries+funding
A. Create new cadre and integrate into MOH staff establishment	- Strong link to health system - Career path - Sustained support - Easier supervision	- Fiscal, administrative and financial constraints.	Zambia – Government of Zambia+GFATM/NGOs
B. Integrate counselling tasks within existing CHW cadre's scope of work	- CHW is accepted cadre - Renewed international and national interest in CHWs	- Increasing job profile without increasing basic training. - May overburden CHWs in settings where they are already multitasked with a “full plate,” poorly remunerated or acknowledged. - Tasks may require additional training. - Work in both facility and community is needed. - Often still donor-funded.	Malawi - Government of Malawi South Africa – DoH and NGOs/CBOs
C. Local/regional government contract	- Local flexibility according to needs - Link to health system	- Dependent on local government priorities. - Works best in mature health system decentralization process. - Vulnerable to hospital budget trends and dependability.	South Africa – DoH and NGOs/CBOs Zimbabwe – GFATM Mozambique – NGOs/donors
D. NGO/CBO contract	- If strong NGO/CBO, quality management - Generally strong connections to patient and community needs - Potential community mobilization to demand and access services	- Vulnerable to donor trends and dependability (domestic funding not usually given to NGOs for this role).	Mozambique – NGOs, GFATM Lesotho – GFATM, NGOs Swaziland – NGOs At the provincial level in South Africa: local NGO combined with government

CHW, community health worker; CBO, community-based organization; DoH, Department of Health; NGO, non-governmental organization; GFATM, Global Fund to fight AIDS, Tuberculosis and Malaria.

#### i) Integration into the government human resources for health establishment

Zambia has successfully integrated psychosocial counsellors into the MOH establishment. Fiscal barriers and wage bill constraints, restricting absorption of new staff into the health systems of countries, often lead to donors continuing to pay for a large number of professional health staff, as is the case in Mozambique, Zimbabwe and Lesotho. There are also administrative constraints on the part of the Ministry of Finance to absorb additional positions allocated to health, and where health budgets are insufficient, professional staff are generally prioritized. In some countries like Mozambique, cadres with a shorter duration of training, including lay counsellors, face constraints in being absorbed as civil servant staff because the system requires at least secondary school as a pre-education level.

#### ii) Integrate counselling into existing CHW cadres

There are clear advantages to integrating counselling tasks within an existing CHW cadre as has been done in Malawi. However, in most assessed countries CHWs are still dependent on international funding. Caution is needed so as not to add too many tasks to an often already broad CHW job profile, while maintaining quality of care. Because CHWs are principally a community cadre, it is important that sufficient numbers be in place to cover both health facility and community-based care.

#### iii) Share responsibility for supporting lay counsellors between the national MOH and local government

Several countries opted for this arrangement, including South Africa, Mozambique and Zimbabwe. In South Africa, lay counsellors working on a local or regional government contract are jointly funded by NGOs and government. Although this option works best when HRH management is decentralized, it is important to centrally define the lay counsellors’ scope of practice as well as having a regulatory framework in place.

#### iv) Employ lay counsellors through community-based organizations and/or NGOs

Lay counsellors are still mostly supported through internationally funded community-based organizations (CBOs) and/or NGOs, as is the case in Lesotho, Mozambique, Swaziland and Zimbabwe. Through their link with civil society, CBOs and NGOs could play a role in supporting community advocacy, connecting patient needs with availability and quality of services. However, linkage with the formal health system is needed to ensure a level of coordination and consistent supervisory support. Because funding mostly comes from external sources, there is a risk of dependence. Costs for the MOH to provide supervision also need to be included in budget calculations. At a time of reduced or flatlined international funding for HIV [45], this is a concern.

## Discussion

This review found considerable variability between countries in defining the role of lay counsellors and the different components of recognition within their health systems. At the start of the HIV epidemic, when awareness around HIV and ART was limited, patient education and counselling focused on increasing general knowledge on HIV and transmission. New areas of counselling needs have evolved over time. For example, with increased access to viral load testing in sub-Saharan Africa came the need for enhanced adherence support, recommended for patients at risk of treatment failure. Adapted counselling is also needed to support the policy shift towards earlier ART initiation, as increasingly people will start treatment without having experienced HIV-related illness. Disclosure counselling for children infected through their mother during pregnancy or delivery a number of years ago is another area that will need to be addressed increasingly. Specific training and on-the-job coaching of lay counsellors is therefore required to ensure they are equipped to address these emerging counselling responsibilities.

In countries where job profiles have not yet been harmonized, clear job descriptions can assist in focusing tasks for lay counsellors, avoiding additional informal responsibilities that could compromise the quality of services provided. For example, in Zambia specialized lay counsellors provide HTS and other related HIV services of decent quality, leading to lower error rates observed in registration and counselling services for lay counsellors in comparison with other health workers [46]. Given the rise in the burden of chronic conditions such as diabetes and hypertension in countries with high HIV prevalence [47] together with HIV co-morbidity, in particular in aging HIV patients [48], integration of HIV and interventions for non-communicable diseases should be considered. Generally, it can be of benefit to draw lessons learned from the management of HIV to address the changing burden of disease [49].

Whilst an integrated approach within an existing cadre has several advantages, a wide variety of responsibilities may impact negatively on the quality of HIV testing [50] and on the feasibility of performing such a wide range of tasks effectively. Research is recommended on the limitations of having multiple tasks included in a lay worker's job profile whilst ensuring quality of care.

In some settings, lay counsellors are recruited amongst PLHIV, such as the lay counsellors in Guinea or Swaziland. Although peers may bring important life experience into counselling support, the feasibility of solely targeting peers as lay counsellors should be considered, especially when lay counsellors are becoming increasingly involved in counselling for other chronic conditions.

The WHO and UNAIDS increasingly recommend community-supported models of care, including HIV testing and alternative ART refill strategies. NGOs are supporting the implementation of these in various countries in sub-Saharan Africa [15,19,51]. In order to support these activities, there is an increased need for lay counsellors to perform some of their activities at the community level [15,19,22]. There are reports of pilot projects that conduct ART initiation and other HIV-related counselling in the community instead of at the health facility [52].

Although there are differences in lay counsellors’ work locations between countries, attention needs to be paid to realistic estimations about workload at both the community and facility levels.

This review found that lay counsellors are increasingly being included in national plans or strategies. However, lay counsellors are mostly not included in national human resources for health data, and information systems therefore need to be strengthened for improved planning to ensure needs are met. Their limited representation in professional associations is another issue that needs to be addressed.

All countries assessed in this review have adopted the recent global 90-90-90 targets and have made subsequent scale-up plans to meet these goals [5,19]. In order to match these ambitions, as well as contribute to the implementation of the new WHO HIV guidelines to initiate all HIV-positive diagnosed people on ART, long-term HRH strategic support and financing are needed. It is therefore important to redefine a national HIV care package and ensure adherence counselling and patient education are included, whilst defining the cadres responsible for these tasks. In countries with insufficient professional staff or where counselling tasks cannot be integrated into existing cadres, lay counsellors could be trained and deployed. In the meantime, long-term strategies such as the creation of a counselling cadre and integrating this in HRH strategic plans can be further developed.

The majority of countries assessed have not yet been able to find ways to sustain lay counsellors or absorb them into their staff establishment. For several reasons, integration of new cadres into the formal MOH staff establishment is challenging. In the first place, increases in health workers are generally limited through the wage bill, a Ministry of Finance-defined ceiling for the country's budget for civil servant staff. Such ceilings were previously set by the International Monetary Fund or the World Bank as a condition for loans, leading to restrictions on hiring staff or improving their remuneration. Since 2007, these ceilings are no longer conditions [53]; however, because they remain recommendations [54,55], ministries of finance still tend to follow them in several countries. For instance, in Lesotho and Mozambique there were serious problems in the recruitment and retention of health staff when freezes in civil servant salaries were put in place. Although countries often have a policy prioritizing health within the overall wage bill, in practice this is mostly not the case [56]. The WHO has also strongly recommended strengthening the health workforce in achieving health targets [33]. The position of the MOH in negotiating their wage bill budget is generally not strong [56]. In addition, international donors only fund staff salaries when a clear plan is presented for long-term sustainability or absorption into the MOH staff establishment [40,57–59]. Another reason for difficulties in absorbing lay cadres and other “non-official” positions is the lack of involvement of the Ministry of Public Service, as reported in several of the assessed countries. In light of these listed difficulties, when integrating more staff into their civil servant establishment, countries naturally prioritize professional healthcare workers over lay cadres, although both are needed.

The lack of published evidence around the cost-effectiveness of lay counsellors has been another constraint for further recognition and financial support. Potential cost savings achieved through improved uptake of services and retention in ART care [13,14], as well as reduced risk of drug resistance through enhanced counselling, need to be documented [60]. More data on cost-effectiveness are needed for MOH to make the case for financing and supporting lay counsellors towards the Ministry of Finance and donors. Due to lay counsellors’ lower remuneration package and training costs, the cadre can be relatively rapidly scaled up compared to professional staff. As an example, PERSAL (the South Africa government staff database) in Western Cape Province reported that 5.3 counsellors can be employed for every professional nurse. In terms of remuneration packages, in Mozambique, the MOH proposes 117 USD per month, being approximately half that of a mid-level nurse, and in Swaziland the package for a lay counsellor is between 100 and 300 USD depending on the supporting partner, compared to 640 USD per month for the lowest level nurse.

Simplified ART delivery strategies, including community-supported models of care, have advantages in terms of cost savings for both patients and health systems and such approaches are recommended by the WHO [61,62]. Lay counsellors can play an important role in the functioning of these models, for example in the formation and facilitation of community ART groups in Mozambique [22]. Reducing the workload for professional health staff allows them to better focus on patients with complex needs that require clinical expertise [18,23,24]. Other study findings confirm similar time-saving benefits for professional staff and therefore cost savings in ART care, making task shifting to lay providers a cost-effective strategy [63].

In Lesotho, in 2013 a rapid workload analysis performed by MSF amongst lay counsellors in three health facilities reported 77 hours per month per lay counsellor, considerably reducing the workload of professional health staff [37]. Adherence clubs, widely implemented in South Africa, where people on ART are able to collect medication and which are facilitated by lay counsellors, were reportedly highly cost-effective compared to conventional nurse-led standards of care [64].

## Conclusions

Patient support activities performed by lay counsellors have demonstrated a positive impact on patient and programme outcomes and will be critical to achieving HIV targets in national strategic plans as well as the ambitious 90-90-90 targets. With the 2015 change in WHO recommendations to start all people with an HIV diagnosis on ART without delay, combined with the fact that only 45% of HIV-positive people know their HIV status, a large increase in testing and treatment is needed. The study findings showed how lay counsellor programmes require adequate support in training, supervision and remuneration in order to function better. Despite lack of clarity in lay counsellors’ scope of practice and limited integration into countries’ health systems, all assessed countries made important steps towards their support and recognition.
